# A machine learning approach uncovers principles and determinants of eukaryotic ribosome pausing

**DOI:** 10.1126/sciadv.ado0738

**Published:** 2024-10-18

**Authors:** Mauricio Aguilar Rangel, Kevin Stein, Judith Frydman

**Affiliations:** Department of Biology, Stanford University; Stanford, CA 94305, USA.

## Abstract

Nonuniform local translation speed dictates diverse protein biogenesis outcomes. To unify known and uncover unknown principles governing eukaryotic elongation rate, we developed a machine learning pipeline to analyze RiboSeq datasets. We find that the chemical nature of the incoming amino acid determines how codon optimality influences elongation rate, with hydrophobic residues more dependent on transfer RNA (tRNA) levels than charged residues. Unexpectedly, we find that wobble interactions exert a widespread effect on elongation pausing, with wobble-mediated decoding being slower than Watson-Crick decoding, irrespective of tRNA levels. Applying our ribosome pausing principles to ribosome collisions reveals that disomes arise upon apposition of fast-decoding and slow-decoding signatures. We conclude that codon choice and tRNA pools are evolutionarily constrained to harmonize elongation rate with cotranslational folding while minimizing wobble pairing and deleterious stalling.

## Teaser

Machine learning aided discovery of mechanisms regulating eukaryotic ribosome translation elongation rate and disome formation.

## INTRODUCTION

Efficient translation of mRNA by the ribosome is central to protein biogenesis. Decoding mRNAs one codon at a time requires matching the correct triplet to its cognate tRNA. The decoding rate for each individual codon in an mRNA can vary markedly, leading ribosomes to move quickly or slowly at specific positions along the transcript (*1*–*3*). This defines a characteristic rhythm of translation elongation which can vary widely for different codon positions in the same mRNA (*3*). It is increasingly recognized that differential local translation rates profoundly affect protein biogenesis. While pausing events have been linked to functionally positive outcomes, including translational fidelity (*4*, *5*), cotranslational protein folding (*6*–*8*), recruitment of nascent chain binding factors (*9*), and other key biological functions, and excessive pausing resulting in ribosomal stalling and collisions is deleterious and activates a number of stress responses (*10*–*12*). Accordingly, understanding the principles that govern local elongation rate has great biological importance, as highlighted by findings that single synonymous codon substitutions that do not change the identity of the incorporated amino acid can lead to loss of protein function and disease [reviewed in (*2*, *13*)].

Various molecular factors and sequence features are known to affect elongation rate; a large body of work implicates contributions of tRNA abundance, mRNA sequence motifs, amino acid chemistry, and features of the nascent polypeptide sequence in elongation pausing [see (*1*, *14*–*16*) for a comprehensive discussion]. tRNA abundance is perhaps the most studied determinant of decoding rate. The genetic code is degenerate, and the same amino acid can be encoded by codons with either abundant or rare cognate tRNAs. Both eukaryotic and bacterial mRNAs exhibit an uneven frequency of codons encoding for the same amino acid, a phenomenon referred to as codon usage bias (*17*–*20*). This bias correlates with the cellular tRNA pool, whereby codons decoded by highly abundant tRNAs are more frequent (*21*, *22*). As highly abundant tRNAs are more readily available to the ribosome, frequent codons decoded by abundant tRNAs are proposed to be translated faster than rare codons decoded by low abundance tRNAs (*7*, *23*). In addition, some cognate tRNAs recognize their codons via wobble pairing interactions in the third codon position. Examples from diverse systems show that such codons decoded via wobble pairing are translated more slowly (*24*–*30*). Recent cryo–electron tomography analysis of the elongation cycle in situ in mammalian cells suggests that accepting an incoming tRNA into the peptidyl transferase A site is the slowest step during elongation in vivo (*31*). This supports the idea that the interaction of the A-site codon with the incoming aminoacyl-tRNA into the ribosome is the critical rate-limiting step of elongation.

The nascent polypeptide also influences the elongation rate. The ribosome can catalyze the formation of all 400 common types of peptide bonds, but not all amino acids are incorporated into the nascent polypeptide chain (NC) at the same rate. For example, the secondary amine and constrained geometry of proline residues slow down their incorporation into the growing NC at the peptidyl transferase center (PTC) of the ribosome (*32*–*34*). The NC itself can also slow elongation via interactions with the ribosome exit tunnel (*35*–*37*). In the first described example, bacterial SecM nascent chains rely on both a proline-induced pause at the PTC and a specific tryptophan-containing sequence in the tunnel to stall elongation (*35*). In addition, stretches of positively charged amino acids slow elongation (*38*–*40*). Possible mechanisms proposed for this slowdown include interactions of the basic nascent chain with the tunnel and π-stacking of poly(A) stretches at the ribosome tRNA binding sites. However, these models cannot adequately explain the near-universal slowdown observed for triplets coding for basic amino acids, which occurs even when there are no basic polypeptide segments in the tunnel or when the basic amino acids are not encoded by AAA codons.

While biochemical, evolutionary, and structural approaches have contributed important mechanistic insight into how tRNAs and amino acids affect elongation rate, the precise role of these factors and their interplay in regulating translation elongation rate in vivo remain poorly understood. The advent of ribosome profiling (*41*) (herein RiboSeq) has helped elucidate features that control translation rate in the cell with unprecedented detail [recently reviewed in (*15*)]. However, the inherently noisy nature of RiboSeq data poses a challenge to the identification of positional information, particularly for lower abundant transcripts having limited coverage. Similar challenges are posed by RiboSeq datasets with low read depth, such as those arising from rare tissues or samples. These limitations have often posed a major hindrance to comprehensively identify translatome-wide mechanistic determinants of ribosome elongation rate from RiboSeq data. In this study, we implement an unsupervised machine learning (ML) pipeline and use it to identify ribosome pausing events in an unbiased manner from a large body of RiboSeq datasets. Our analysis enabled us to develop an integrated framework that describes the principles and determinants of eukaryotic ribosome pausing and collisions.

## RESULTS

### Unsupervised ML enables pause site detection despite heterogeneous nature of RiboSeq data

**Fig. 1.**
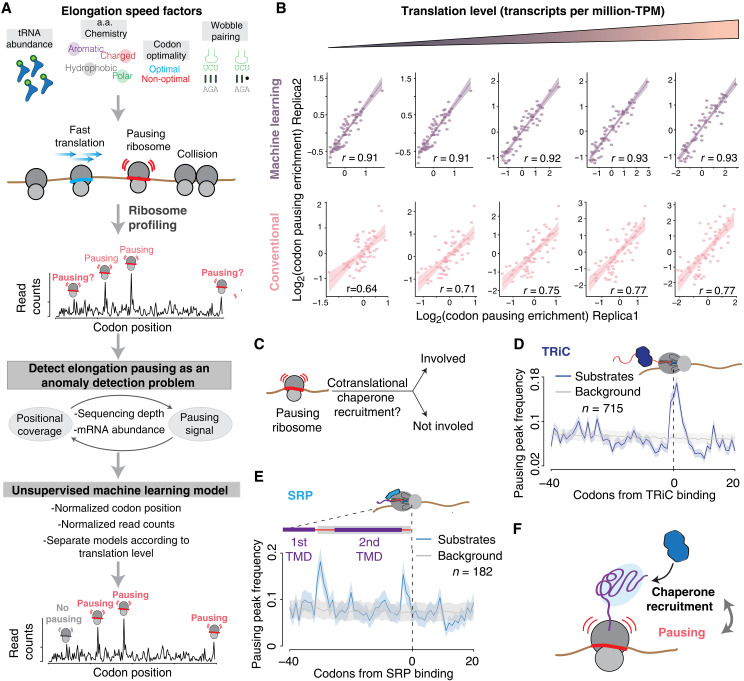
Unsupervised ML enables the identification of transcriptome-wide ribosome pause sites and their correlation with cotranslational folding events. (**A**) Strategy used for the mapping of pausing peaks in RiboSeq and DisomeSeq data (a.a., amino acid). (**B**) Performance comparison between the ML approach described in (A) and a conventional *z* score–based pausing identification method. Scatterplots correspond to different levels of translation based on TPM quantiles {from left to right: (0 to 20%), [20 to 40%), [40 to 60%), [60 to 80%), and [80 to 100%]}; each dot represents a codon. (**C**) Schematic depicting the possible involvement of ribosome pausing in cotranslational folding. (**D** and **E**) Metagene analysis of pausing peak frequency at the moment of chaperone binding (D, TRiC; E, SRP) to the NC. Background is estimated from random positions in the yeast proteome. (**F**) Representation of the functional roles of ribosome pausing during protein biogenesis.

Translatome-wide analysis of elongation pausing using RiboSeq is complicated by the noisy nature of the data and the fact that sequencing coverage decreases with mRNA translation levels. Analyzing RiboSeq data with conventional next generation sequencing (NGS) pipelines creates spurious signals and leads to a bias toward describing the behavior of abundant transcripts and highly translated genes. To address these challenges, we treated the mapping of ribosome pausing sites within RiboSeq data as an anomaly detection problem depending on two variables: position (codon number) and pausing intensity (coverage) (Fig. 1A). To detect anomalies (i.e., true pausing events), we used the extended isolation forest (EIF) algorithm, an unsupervised ML ensemble method which assigns an overall anomaly score to each observation based on an ensemble of scores (*42*, *43*). This offers the advantage of every codon being compared hundreds of times against a random partition of the translatome, instead of a single translatome or gene-wide comparison as done in traditional pausing calculations, such as *z* scores. To identify pausing sites across the entire translatome of the budding yeast *Saccharomyces cerevisiae*, we partitioned two replicate RiboSeq datasets into five categories according to the translation level and trained an EIF on each. To validate our strategy, we calculated the enrichment of codons in each set of identified pausing sites and assessed the correlation across replicates. Our approach proved to be highly consistent across replicates (*r* > 0.9) regardless of mRNA translation levels (Fig. 1B). We compared these results with those obtained applying more common *z* score–based approaches (*44*–*47*) to identify pausing sites in the same RiboSeq datasets. The *z* score–based approach yielded poorer correlations between replicates and a clear coverage-dependent bias (Fig. 1B). Analysis of simulated ribosome pauses in RiboSeq data resulted in an area under the receiver operating characteristic curve (AUC) = 0.98, with a true positive rate of 0.96 and a false positive rate of 0.09 (fig. S1, A and B, and Materials and Methods), further validating our strategy for pausing identification. These analyses demonstrate the power of our strategy to consistently identify pausing sites in RiboSeq data in a translatome-wide manner, even for low abundance transcripts.

To further assess the performance of our approach, we next examined the overlap of identified pausing peaks for each translation level category and across replicates. Overall, there was a conservative overlap between replicates (~20%; fig. S2A), which increased with sequence coverage and was highest for highly translated transcripts (fig. S2B). Despite the conservative overlap, the same codon features determined pausing in each of the translation level–based partitions, as all codons displayed virtually the same level of enrichment at pausing sites (all *r* > 0.9; fig. S2C). The strong correlation between codons enriched at pausing sites for both high and low-coverage sets of mRNAs confirms that these identified positions do not constitute a random group of codons but are bona fide pausing sites. Further confirming this conclusion, the correlation between codons enriched in pausing sites was maintained when comparing replicate overlapping versus replicate nonoverlapping pausing sites but was lost in a randomized control set of translatome positions (fig. S2, D and E). The modest overlap between pausing sites in both replicates is driven by lower-coverage open reading frames (ORFs) (fig. S2B) due to the more heterogeneous coverage distribution in low translated ORFs compared to a more uniform coverage in high translated ORFs. The ability to detect pausing events in low-coverage data and low-expression genes reveals that elongation pausing events are driven by consistent mechanisms across the translatome regardless of gene expression levels. We conclude that our ML approach can facilitate the comprehensive translatome-wide identification of ribosome pausing sites, independent of mRNA expression levels, to define determinants of elongation rate. By enabling analysis of low-expression and low-coverage RiboSeq datasets, our ML approach should help understand translation dynamics in rare samples or single-cell RiboSeq analyses.

### Elongation pauses are linked to biological signatures of cotranslational proteostasis

Previous analyses of cotranslational recruitment of chaperones and targeting factors to NC found a correlation between recruitment events and translation of codons with low translation adaptation index (tAI; a proxy for codon optimality) (*6*, *9*). To test the hypothesis that elongation pausing facilitates cotranslational recruitment of proteostasis factors, we next used a reciprocal analysis, whereby we examined whether the pausing events identified with our ML pipeline correlated with the previously mapped binding of two distinct cotranslationally acting factors: the chaperonin TRiC/CCT and the endoplasmic reticulum (ER)–targeting factor signal recognition particle (SRP) (Fig. 1C) (*48*, *49*). These analyses, which circumvent the need to rely on a single pausing metric such as tAI, also validated the ability of our approach to identify biologically relevant pausing events.

The chaperonin TRiC/CCT binds primarily cytonuclear proteins, while the ER-targeting factor SRP binds secretory pathway proteins (Fig. 1C) (*48*, *49*). Despite being recruited to different subsets of nascent chains, our analysis comparing chaperone binding events against ribosome pausing indicates that the cotranslational binding events for both proteostasis factors are strongly enriched in ribosome pausing sites (Fig. 1, D and E). We observe that for TRiC substrates, the recruitment event occurs in concert with a pausing site (Fig. 1D). For SRP substrates, the pausing events occurred immediately after the transmembrane domain (TMD) acting as a signal sequence is translated, which in turn correlated with SRP recruitment (fig. S3A). This was observed regardless of the number of TMDs in the protein (Fig. 1E and fig. S3B). Furthermore, TMDs not serving as SRP recruitment sites were not enriched in pausing sites (fig. S3C). These results validate that our ML approach can identify pause sites linked to cotranslational recruitment of proteostasis factors.

Since our analysis of pausing sites is agnostic to mechanism, we next examined whether specific pausing elements govern these events. Notably, SRP and TRiC binding events are both mediated by pausing events that differentially use low tAI and proline-mediated pausing mechanisms. Thus, association of both factors with nascent chains was enriched by pausing relying on prolines and local decreases in tAI. However, SRP binding was more strongly associated with decrease in tAI than TRiC binding, with the latter being about half the magnitude of the tAI change observed for SRP (fig. S3, D and F). In contrast, the proline presence was stronger at the time of TRiC-binding events, with two ribosome sites displaying strong proline enrichment during TRiC binding and only one site for SRP binding events (fig. S3, E and G).

These analyses support the concept that elongation pausing promotes cotranslational nascent chain recognition by proteostasis factors and raises the possibility that different factors use distinctive pausing mechanisms. Collectively, these results demonstrate that the ribosome pausing sites identified through our unsupervised ML approach are linked to biologically meaningful cotranslational folding signatures.

### A complex interplay between codon optimality and amino acid properties determines pausing along the elongation cycle

**Fig. 2.**
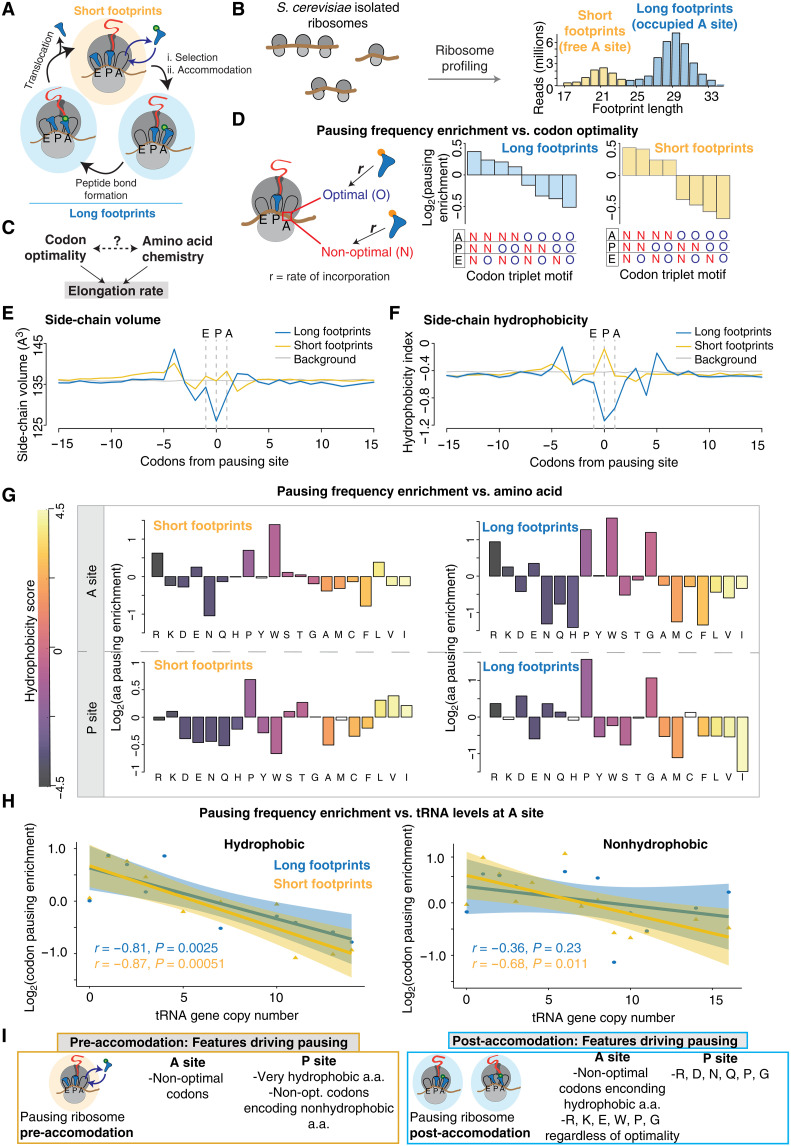
Dissecting the role of amino acid properties and tRNA abundance on elongation pausing. (**A**) The different translation elongation phases and their distinctive ribosome profiling footprint lengths. (**B**) Example of the short and long ribosome footprint populations generated from isolated ribosomes. (**C**) Variables affecting translation elongation rate. (**D**) Enrichment analysis of the codon optimality motifs located in the ribosome tRNA binding sites when a pausing peak is located in the A site. (**E**) Metagene analysis of side-chain volume occupancy in the regions flanking pausing sites. (**F**) Metagene analysis of hydrophobicity in the regions flanking pausing sites. (**G**) Amino acid enrichment at the P and A sites of pausing ribosomes (odds ratio by Fisher’s exact test, white bars denote nonsignificant scores). (**H**) The correlation between codon enrichment in the A site during a ribosome pausing event and their corresponding number of decoding tRNA gene copies, separated according to amino acid hydrophobicity. (**I**) Summary of the derived pausing principles.

The ribosome contains three tRNA binding sites, referred to as A, P, and E (Fig. 2, A and B). During the elongation cycle, the incoming amino-acyl tRNA (aa-tRNA) is accepted into the A site and accommodated into the ribosome, the P site binds the growing peptidyl-tRNA, and the E site binds the uncharged tRNA as it exits the ribosome (*50*). Pausing in the distinct ribosomal states transited during this elongation cycle can be captured in RiboSeq experiments via analyses of short and long footprint populations (Fig. 2, A and B). Short footprint reads arise from ribosomes that have not yet accommodated an incoming aa-tRNA and thus have an empty A site, whereas long reads arise from post-accommodation ribosomes with an occupied A site (Fig. 2A) (*51*, *52*). Since distinct stages of the elongation cycle may be associated with different rate-limiting determinants, we applied our ML pipeline to identify pausing events in the distinct ribosome conformations giving rise to long and short RiboSeq reads.

Among the several factors influencing translation decoding rate, codon optimality, tRNA abundance (*7*, *23*), and amino acid chemistry (*53*) exhibit an unclear relationship (Fig. 2C). To understand how these factors influence pausing at different parts of the elongation cycle, we first examined the role of each variable in pausing events in long and short reads independently, and then we examined whether there is synergy or hierarchy for tRNA abundance and amino acid identity in determining pausing.

First, we examined the contribution of codon optimality (see Materials and Methods) to elongation pausing. Previous work using translation inhibitors to avoid artifactual short footprints showed a strong overall positive correlation between A-site centered short reads and low codon optimality (*52*). This correlation was weaker for long footprints. We applied our analysis pipeline to these previous datasets (*52*) to identify ribosome pause sites in long and short reads and examine their relationship with codon optimality. To this end, we calculated the enrichment of all possible codon triplets formed by optimal (O) and non-optimal (N) codons that are occupying the A, P, and E sites of the pausing ribosome (Fig. 2D). Notably, codon optimality exerted a hierarchical and position-dependent impact on elongation kinetics depending on the tRNA binding sites and the state of the ribosome. As expected, triplets of optimal codons occupying the three ribosome tRNA binding sites (OOO) were depleted from pausing sites, indicating that these sites are rapidly translated. By contrast, non-optimal codons simultaneously occupying A, P, and E sites (NNN) were most enriched in pauses observed in both long and short read populations. The effects of codon optimality were more pronounced in the short read pausing sites, but in both cases, the presence of non-optimal codons in the A site was the major determinant of pausing, followed by non-optimal codons in the P site, and lastly, non-optimal codons in the E site (Fig. 2D). This analysis reveals a nuanced role of codon optimality on elongation pausing, indicating that pausing is a function of both optimality and position within the ribosome’s active site.

Next, we examined how physicochemical properties of amino acids in the A and P sites of the PTC influence elongation pausing. We observed that side-chain volume and hydrophobicity primarily influence ribosome pausing in the post-accommodation state, represented by long footprints (Fig. 2, E and F). During pausing, residues with smaller side-chain size were significantly enriched at the PTC, whereas bulkier residues were enriched in the PTC-proximal part of the exit tunnel (Fig. 2E). Breaking down those analyses by residue, proline stood out as the most commonly overrepresented residue in the P and A sites for both pre- and post-accommodation states as expected from previous studies (Fig. 2G). The amino acid enrichment trends in the A site were similar for the pre- and post-accommodation states, with proline, tryptophan, arginine, and glutamic acid enriched in pausing sites and most hydrophobic side chains largely depleted (Fig. 2G). Notably, glycine, lysine, and leucine were differentially enriched in long and short reads, suggesting that these side chains present distinct challenges pre- and post-accommodation (Fig. 2G). The physicochemical properties of amino acids occupying the P site of pre- and post-accommodation ribosomes displayed different and often opposing trends. The P site of ribosomes paused in the pre-accommodation state exhibit a moderate enrichment of highly hydrophobic side chains (L, V, and I) and a clear depletion of nonhydrophobic residues, whereas the P site of ribosomes pausing in the post-accommodation state were depleted of hydrophobic residues, except for glycine, and enriched in several nonhydrophobic ones (Fig. 2G). These distinct amino acid enrichments in the A and P sites of paused ribosomes likely reflect the chemical constraints of peptide bond formation and tRNA incorporation and movement along the elongation cycle.

We next explored the interplay and synergy between codon optimality and amino acid properties in determining elongation rate. As codon optimality is a function of the tRNA abundance in the cell, we started by analyzing the relationship between tRNA copy number and amino acid hydrophobicity in promoting elongation pausing. We classified codons based on whether they encode for hydrophobic or nonhydrophobic residues and then examined separately their enrichment at pausing sites as a function of tRNA gene copy number. As expected from the above analysis, pausing at pre-accommodation ribosomes exhibited a strong negative correlation between tRNA copy number and A-site codon enrichment regardless of amino acid properties (*r* = −0.87 for hydrophobic and −0.68 for nonhydrophobic amino acid encoding codons) (Fig. 2H). Unexpectedly, pausing in ribosomes in the post-accommodation state evinced a complex interplay between codon optimality and amino acid chemistry. Examining A-site codons in paused post-accommodation ribosomes revealed that low tRNA levels correlated well with pausing enrichment when the codons encoded hydrophobic residues (*r* = −0.81), but there was no significant correlation with tRNA levels when the A-site codons encoded nonhydrophobic amino acids (*r* = −0.36; Fig. 2H). It thus appears that the contribution of A-site codons to pausing after tRNA selection, i.e., during and after accommodation, is driven by tRNA levels when the codons encode hydrophobic amino acids but is driven by side-chain properties when nonhydrophobic residues are encoded (Fig. 2G).

We applied a similar analysis to link codon optimality and amino acid properties to pausing for P-site codons in both pre- and post-accommodation states (Fig. 2G and fig. S4B). In contrast to the A site, there were only weak and mostly nonsignificant associations between tRNA levels and pausing from both pre- and post-accommodation states (fig. S4B). This indicates that the major contributions of P-site codons to pausing arise from amino acid chemistry with only minor contributions of tRNA levels. Together, our analysis reveals that ribosome pausing is shaped by a complex interplay between codon optimality and amino acid size and hydrophobicity in both P and A sites, which influences in disparate ways the pre- and post-accommodation steps of the translation elongation cycle (Fig. 2I).

### Altering tRNA levels reveals widespread role of wobble pairing in decoding the translatome

Similar to the interplay between tRNA levels and amino acid chemistry, previous work has shown that wobble pairing between tRNA and mRNA can affect the rate of translation (*21*). Because the genetic code contains more codons than the available decoding tRNA isoacceptors, some codons must be decoded with a tRNA that recognizes the third codon position through wobble interactions (*54*). In vitro translation studies and ribosome profiling show that these wobble codons are decoded more slowly than direct interactions (*39*). Certain codon pairs containing wobble pairing elements result in major translation slowdowns (*29*), which is alleviated to a greater extent by expressing non-native, exact-matching tRNAs than by increasing the levels of the endogenous wobble-decoding tRNAs (*29*). However, we have a limited understanding of how the relationship between tRNA abundance and wobble pairing affects codon decoding rates in vivo. The biological significance of understanding this relationship is highlighted by studies showing that altering tRNA levels in yeast and bacteria impair proteostasis and lead to aggregation of misfolded proteins (*55*–*57*).

**Fig. 3.**
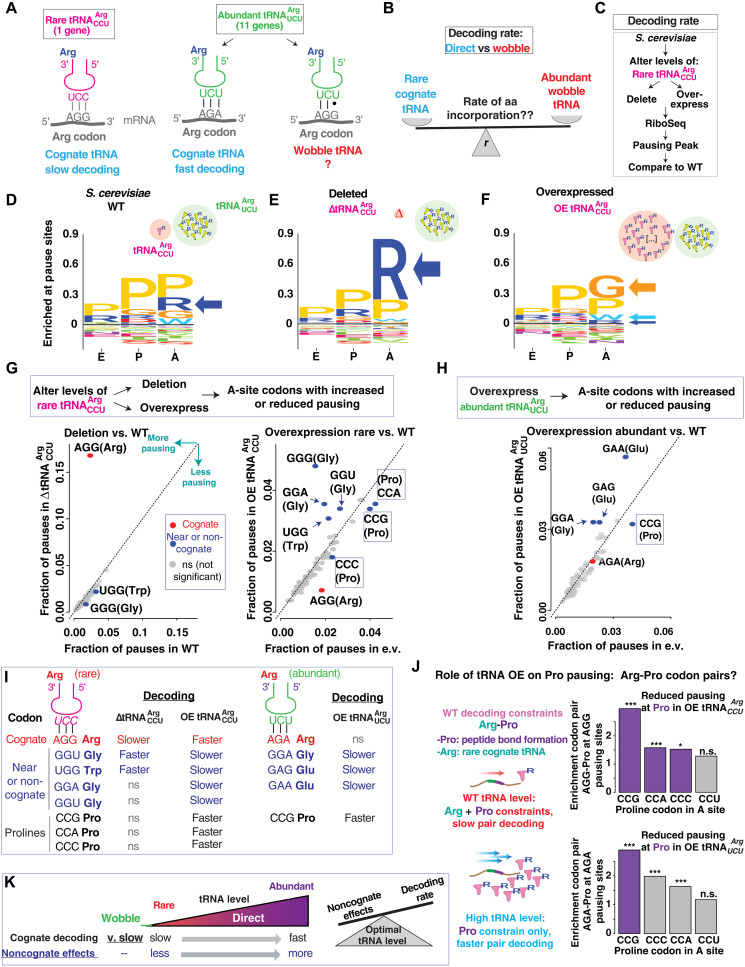
Altering tRNA levels reveals widespread role of wobble pairing in decoding the translatome. (**A**) The two known decoding mechanisms for the rare AGG-Arg codon in *S. cerevisiae*. (**B**) Comparing the impact of tRNA abundance and wobble pairing on decoding the AGG codon. (**C**) Flowchart of the strategy used to test the effects of altering the tRNAArgCCU levels in *S. cerevisiae*. (**D** to **F**) Logo plot analysis of the identified pausing peaks for the WT *S. cerevisiae*, the ΔtRNAArgCCU, and the tRNAArgCCU overexpression strains (pausing peak centered at the A site). (**G**) Scatterplot comparing the fraction of pausing sites for each codon: WT versus ΔtRNAArgCCU (left) and WT plus empty vector versus tRNAArgCCU over-expression (right). Blue and red dots represent the outliers identified by a Theil-Sen regression (OE, overexpression, e.v., empty vector). (**H**) Scatterplot comparing the fraction of pausing sites for each codon in WT plus empty vector versus tRNAArgUCU overexpression. Blue dots represent the outliers identified by a Theil-Sen regression. (**I**) Summary of the observed effects that deleting or overexpressing rare and abundant tRNAs has on cognate and noncognate codons. (**J**) Enrichment analysis of AGG-CC(A/C/G/U) (top) or AGA-CC(A/C/G/U) (bottom) codon pairs at AGG or AGA pausing sites, respectively, in the empty vector background from (F) and (H). Coloring of the bars corresponds to the coloring in (F) (right) and (H); asterisks indicate statistical significance of the odds ratio (Fisher’s exact test). (**K**) Schematic depicting the relationship between tRNA decoding mechanism (cognate interaction versus wobble pairing) and tRNA abundance in codon decoding rate. Changes in pausing in this figure are derived from two biological replicates.

To study how the interdependence of wobble pairing and tRNA levels affects elongation pausing, we applied our RiboSeq pipeline to yeast with altered levels of either the rarest or the most abundant Arg-decoding tRNAs. In *S. cerevisiae*, the AGG codon for arginine is preferentially decoded by the rare single-copy tRNA^Arg^_CCU_ isoacceptor (Fig. 3, A and B). In the absence of its exact matching tRNA pair, AGG is decoded via wobble pairing by the abundant tRNA^Arg^_UCU_ isoacceptor, which has a gene copy number 10-fold greater than tRNA^Arg^_CCU_ (Fig. 3A) (*55*). In wild-type (WT) cells, the AGG codon could be decoded in principle either directly by a rare cognate tRNA that forms Watson-Crick (WC) base pairs at all three codon positions, tRNA^Arg^_CCU_, or through slower wobble pairing decoding by the abundant tRNA^Arg^_UCU_. We reasoned that altering the levels of the single gene copy tRNA^Arg^_CCU_, by either deletion or overexpression, would illuminate the relative contributions of tRNA availability and wobble pairing to ribosome pausing (Fig. 3, B and C). If tRNA abundance takes precedence over wobble pairing in determining elongation rate, then deleting *tRNA^Arg^_CCU_* should not affect the elongation pausing profile of cells relative to WT (Fig. 3D) because the AGG codons would be readily decoded by the abundant tRNA^Arg^_UCU_ species.

To test the above hypothesis, we either deleted (Fig. 3E) or overexpressed (Fig. 3F) tRNA^Arg^_CCU_ as described (*55*). We then carried out RiboSeq analyses to identify sites of ribosome pausing, followed by analysis of codon and amino acid enrichment in the P and A sites of the ribosome (Fig. 3C). Upon deletion of tRNA^Arg^_CCU_, we observed a drastic enrichment in A-site pausing at only AGG codons in Δ*tRNA^Arg^_CCU_* cells compared to WT as expected from increased pausing (Fig. 3G, left). This suggests that decoding via wobble pairing, even by an abundant tRNA, is much slower than WC decoding in the third codon position by a rare tRNA and could not compensate for loss of tRNA^Arg^_CCU_.

In addition to increased pausing at AGG codons, deleting *tRNA^Arg^_CCU_* caused a small but statistically significant decrease in pausing at codons GGG and TGG, encoding glycine and tryptophan, respectively (Fig. 3G, left), which are near cognates of the AGG (Arg) codon, differing only in the first position. The faster translation at these codons in the absence of tRNA^Arg^_CCU_ reveals that even rare tRNAs, such as the single gene copy tRNA^Arg^_CCU_, can interfere with decoding of near-cognate codons and cause a slowdown in their decoding by their cognate tRNAs. This is consistent with previous in vitro and in silico results suggesting near and noncognate aa-tRNAs can compete with cognate aa-tRNAs (*58*, *59*).

To further explore the relationship between tRNA abundance and wobble pairing on ribosome pausing, we tested the converse effect by performing RiboSeq of cells overexpressing the rare tRNA^Arg^_CCU_ (Fig. 3F). We hypothesized that increased levels of cognate tRNA^Arg^_CCU_ should outcompete wobble decoding by tRNA^Arg^_UCU_ and thus decrease even further ribosome pausing caused by AGG codons occupying the A site. Overexpression of tRNA^Arg^_CCU_ reduced pausing at AGG codons compared to WT cells. Of note, this experiment led to two unexpected observations (Fig. 3G, right). First, we observed that tRNA^Arg^_CCU_ overexpression exacerbated ribosome pausing at several codons: at near-cognate codons UGG and GGG, which satisfyingly were the same codons that exhibited less pausing in Δ*tRNA^Arg^_CCU_* cells (Fig. 3G), and at two noncognate glycine codons, GGA and GGU (Fig. 3G).

To obtain independent validation of the above conclusions, we next overexpressed an abundant arginine tRNA, namely tRNA^Arg^_UCU_, which exists in excess supply relative to its cognate AGA codon according to a supply-demand model of translation (*6*). We reasoned that increasing the levels of this isoacceptor should not reduce the frequency of ribosome pausing at AGA codons but could affect decoding of near-cognate codons. Overexpression of tRNA^Arg^_UCU_ did not alter pausing during AGA decoding (Fig. 3H), reaffirming the tRNA^Arg^_UCU_ surplus relative to AGA codon usage that would be available to decode AGG codons in the Δ*tRNA^Arg^_CCU_* strain. Overexpressing tRNA^Arg^_UCU_ led to an unexpected increase in ribosome pausing for three codons that are either near-cognate (GGA) or noncognate (GAA and GAG) to tRNA^Arg^_UCU_ (Fig. 3H). Thus, increasing the levels of this abundant tRNA leads to a wobble pairing effect on noncognate decoding, similar to that observed when the low abundant tRNA^Arg^_CCU_ is overexpressed (Fig. 3I).

Another interesting observation was that overexpression of tRNA^Arg^_CCU_ decreased pausing at three of the four proline codons (Fig. 3G, right), and overexpression of tRNA^Arg^_UCU_ decreased pausing at one of the proline codons (Fig. 3H). To understand this, we first hypothesized that higher tRNA^Arg^_CCU_ levels could affect the translation rate at arginine-proline pairs encoded by AGG-CC(G/A/C/U) bicodons. Under this scenario, WT cells elongating these codon pairs would face the synergistic challenges of low availability of tRNA^Arg^_CCU_ and inherently slow peptidyl bond formation with a proline. To test this, we examined the frequency of AGG pausing, in WT cells, when paired upstream of each of the proline codons. We found that CC(G/A/C) proline codons exhibiting reduced pausing in tRNA^Arg^_CCU_ overexpressing cells are more frequently found in the A site when an AGG pausing codon is in the P site, while the unaffected proline CCU codon was not enriched in AGG-CCU pausing (Fig. 3J, top). Although arginine-proline pairs encoded by AGA-CC(G/A/C) bicodons are overrepresented at AGA pausing sites in the WT translatome (Fig. 3J, bottom), only the Pro codon in the most overrepresented pair showed faster decoding upon tRNA^Arg^_UCU_ overexpression (Fig. 3J, bottom). This could be explained if the levels of the abundant tRNA^Arg^_UCU_ suffice to overcome the tRNA availability constrain on decoding rate for the less prevalent AGA-CC(A/C/U) pairs but are more limiting for the more abundant AGA-CCG pair. Furthermore, performing a similar analysis with the inversely ordered bicodons, i.e., CC(G/A/C/U)-AG(A/G), shows mostly nonsignificant effects, with only two of eight pairs displaying a very small change (fig. S5).

Together, our results reveal how changing the levels of a single tRNA have far-reaching effects on the decoding rates of near-cognate and even noncognate codons that encode amino acids with very different properties (Fig. 3K). They indicate that individual codon decoding rates in vivo are determined by an intricate relationship between the levels of multiple tRNA isoacceptors and not just by the levels of its cognate tRNA (Fig. 3K).

### Different PTC signatures of elongation pausing are linked to distinct nascent chain folding elements and exit tunnel motifs

**Fig. 4.**
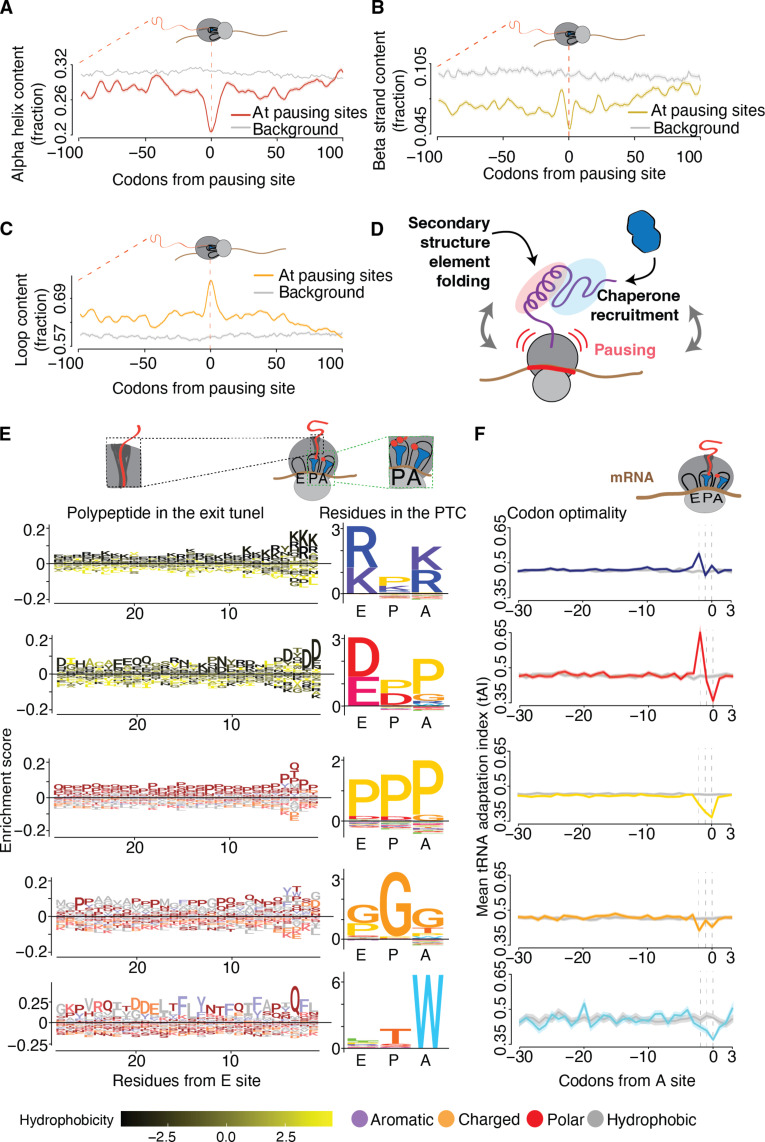
Linking elongation pausing to nascent chain folding elements and exit tunnel sequence motifs. (**A** to **C**) Metagene analysis of protein secondary structure content: α helix (A), β sheet (B), and loop (C) at the moment of ribosome pausing. Negative numbers correspond to translated codons, whereas positive numbered codons have yet to be decoded. (**D**) Pausing promotes proteostasis, secondary structure folding, and chaperone binding. (**E**) Logo plots of the PTC tripeptide clusters identified during ribosome pausing (right side plots) and the associated residues located in the ribosome exit tunnel (left side plots). (**F**) Corresponding tAI traces for the amino acids analyzed in (E).

As translation progresses, the NC leaves the PTC and starts traversing the narrow ribosome exit tunnel, where secondary structure elements can start to form (*60*). Given the link between pausing and cotranslational factor binding (Fig. 1, C to F), we tested whether ribosome pausing also correlates with secondary structure element folding in the nascent chain. We examined the secondary structure propensities of the nascent chain encoded in regions of the translatome proximal to pausing sites in at least three consecutive codons occupying the A, P, and E sites of the ribosome (Fig. 4, A to C). While α helix (Fig. 4A) or β strand propensity (Fig. 4B) were depleted in NC proximal to a pausing site, there was a marked enrichment in regions of loop content (Fig. 4C). These analyses indicate that pausing events mediate the transition between large unstructured, loop-rich regions and segments with higher α helix or β strand propensity (Fig. 4, A to C). We propose that pausing events regulate cotranslational chaperone recruitment to NC in coordination with folding of secondary structure elements upon emergence from the ribosome exit tunnel (Fig. 4D) (*6*).

We next examined whether specific NC sequence motifs are linked to elongation pausing. Previous work, primarily in bacteria, shows that interactions between the NC and the exit tunnel, such as stretches of positively charged residues (*40*, *61*, *62*) or the presence of arrest peptide sequences, folded and unfolded (*37*), contribute to ribosome pausing. Supporting the notion that ribosome pausing may not depend solely on the elements in the A, P, and E sites, it has been shown that some proline-induced pausing events depend on the NC context (*63*, *64*). Therefore, we explored the possible connection between ribosome pausing and the properties of the NC occupying the ribosomal exit tunnel. We started by clustering, by sequence similarity, the tripeptide sequences enriched in the codons occupying the A, P, and E sites during pausing. This identified five major distinct clusters for E-P-A sequences linked to pausing (Fig. 4E), which included specific tripeptide motifs with distinct chemical properties, including a positively charged amino acid triplet, a negatively charged and proline amino acid triplet, a polyproline and a polyglycine tripeptide, as well as a hydrophobic motif with a tryptophan in the A site. While primarily driven by amino acid properties, pausing at some of these tripeptide motifs were also driven by non-optimal codon enrichment in the A site (Fig. 4F).

We then evaluated whether the residues upstream of each PTC cluster, which would occupy the ribosomal exit tunnel, displayed any particular amino acid biases. Notably, the nascent chains occupying the ribosomal tunnel upstream of all these motifs were enriched in specific types of amino acids, with the more PTC-proximal residues displaying a stronger enrichment (Fig. 4E), in agreement with previous findings (*65*, *66*). For the two clusters containing charged residues in the PTC, namely the positive tripeptide cluster and negative-proline tripeptide cluster, the NC occupying the tunnel was significantly enriched in similarly charged residues (i.e., positively or negatively charged, respectively), especially in the preconstriction residues closer to the PTC. For the poly-proline cluster, the NCs in the tunnel were enriched in noncharged polar residues, such as glutamines and threonines as well as prolines. Again, the enrichment was highest in sequences occupying the preconstriction region of the tunnel at the time of pausing. In turn, NCs in the exit tunnel upstream of the poly-glycine PTC motif pause sites were enriched in hydrophobic residues and depleted of charged residues. Last, the cluster characterized by the presence of tryptophan in the A site showed a bias for aromatic residues ~16 residues into the NC immediately upstream of the pause site, corresponding to residues occupying the preconstriction and constriction regions of the tunnel. To make sure these amino acid enrichment trends in the NCs occupying the tunnel were not confounded by codon optimality effects when they themselves were decoded, we calculated the mean tAI along the sequences encoding the NCs. Only the codons in the A, P, and E sites exhibit tRNA abundance signatures, whereas none of the sequences encoding the NC within the tunnel deviate from the background proteome tAI trace (Fig. 4F). This agrees with our finding that the interplay between amino acids and codon optimality at the PTC determines pausing (Fig. 2) and further suggests that the amino acid sequence in the tunnel and not codon optimality at decoding contributes to pausing. We conclude that the interplay between PTC signatures and the NC properties in the exit tunnel regulates ribosome pausing during eukaryotic translation, which may be more prevalent than previously thought.

### Applying translation pausing principles reveals that ribosome collisions arise at disomes translating apposed fast and slow codons

**Fig. 5.**
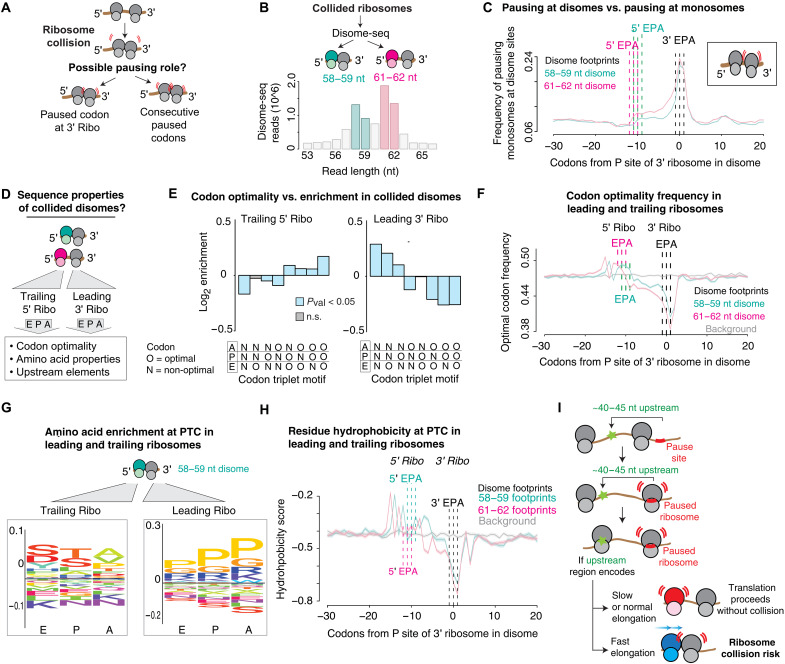
Translation pausing principles help explain ribosome collisions. (**A**) Possible roles of ribosome pausing in disome formation. (**B**) Barplot of sequencing depth by read length in the analyzed disome-seq datasets. Two disome footprint species are considered separately (teal and pink bars). (**C**) Metagene analysis representing the frequency of pausing peaks identified in monosome-derived data at the positions where a disome pausing peak was identified (centered at the P site of the 3′ trailing ribosome of the disome). Inset represents the most likely scenario of disome formation involving ribosome pausing. (**D**) Schematic depicting the possible sequence features influencing disome formation. (**E**) Enrichment analysis of the codon optimality motifs located in the E, P, and A sites of the leading (5′) and trailing (3′) units of the identified disome sites. (**F**) Metagene analysis of the frequency of optimal codons at positions flanking the E, P, and A sites of the identified disome pausing peaks. (**G**) Logo plot analysis of the amino acids located in the PTC of the trailing and leading units of the identified disomes. (**H**) Metagene analysis of the hydrophobicity scores at positions flanking the E, P, and A sites of the identified disome pausing peaks. (**I**) Model representing the mechanism by which disomes form via ribosome collisions involving a single leading pausing ribosome and a trailing ribosome traversing a region of fast translation.

We next applied our unsupervised ML approach to examine how pausing can go awry and lead to ribosome collisions. Disomes formed by collided ribosomes are sensed by specialized quality control pathways that activate stress responses and promote degradation of the mRNA and the nascent polypeptides from the resulting disome [recently reviewed in (*67*)].Our understanding of ribosome collisions has been primarily informed by either stalling reporters containing repeats of slowly decoded codons or in vitro translation reactions of endogenous mRNAs (*68*–*71*). While this has greatly illuminated various mechanistic aspects of disome formation, the sequence-level principles and determinants that cause a ribosome slowdown to turn into a ribosome collision in the cell remain unclear (Fig. 5A). Recent studies identified sites of ribosome collision in eukaryotic translatomes by exploiting the observation that collided disomes protect a larger stretch of mRNA compared to a single ribosome (*72*–*75*). This enabled development of disome profiling (disome-seq) as an extension of RiboSeq by isolating these characteristically longer mRNA footprints (Fig. 5B) (*73*–*75*).

Not every paused ribosome leads to collided disomes. To identify events and determinants leading to ribosome collisions as compared to ribosome pausing, we applied our EIF pausing identification pipeline to disome-seq datasets (*73*). Since disome-seq produces two distinctive read-length populations separated by about three nucleotides, we generated separate models for each of these populations to ensure precise ribosome assignment along the transcript (Fig. 5B) (*73*). We also used RiboSeq data of monosomes to examine the frequency of monosome pausing at both the leading and lagging ribosome positions identified at the disome peak sites. By assimilating these datasets, we found that the leading ribosome shows a canonical pausing profile, with a pausing peak occurring at the E, P, and A sites of the leading ribosome (Fig. 5C). In contrast, the trailing ribosome showed negligible pausing at those sites in monosome RiboSeq data (Fig. 5C). Notably, these pausing profiles were consistent for both read-length populations, with the only difference being a one-codon displacement in the pausing peak that agrees with the one-codon difference in read length. These data indicate that disomes are not the product of two consecutively juxtaposed ribosome pausing sites. Instead, the analysis indicates that pausing elements in disomes formed naturally in vivo are confined to the leading ribosome.

We then used the ribosome pausing principles learned from our above analyses to explore the features that characterize disome peaks and uncover the determinants of ribosome collisions. To identify these determinants, we examined sequence context of both the leading and the trailing ribosome in the collided disome (Fig. 5D). First, we explored the impact of codon optimality in the tRNA binding sites of both ribosomes of the disome. As expected, the E, P, and A sites in the leading ribosome are generally enriched in stretches of non-optimal codons, characteristic of a paused monomeric ribosome (Fig. 2D). By contrast, the E, P, and A sites of the trailing ribosome were enriched in stretches of optimal codons (Fig. 5E). These distinct patterns between the leading and trailing ribosomes agree with our observation that only the leading ribosome experiences a notable translation slowdown across the transcript population (Fig. 5C). To confirm these observations, we performed a metagene analysis of the frequency of optimal codons in the regions surrounding the E, P, and A sites of each ribosome in a disome (Fig. 5F). Unlike the leading ribosome, the E, P, A, and upstream sequences of the trailing ribosome were enriched in optimal codons. In particular, the regions immediately upstream of those in the E, P, and A sites of the trailing ribosome had a higher tendency to contain optimal codons, whereas the codons surrounding the tRNA binding sites of the leading ribosome were highly non-optimal (Fig. 5F).

We next examined the chemical properties of the encoded amino acids in the PTC for each ribosome in the disome. The codons in the PTC of the leading ribosome were strongly enriched in pause-inducing residues such as Pro, Lys, or Arg. This trend mirrored the enrichment in pause-inducing non-optimal codons in the E, P, and A sites of the leading ribosome. In contrast, the PTC of the trailing ribosome, as well as sequences upstream, was enriched in codons corresponding to more rapidly decoded hydrophobic and polar residues (Fig. 5, G and H), again resonating with the enrichment in optimal codons in the E, P, and A sites of the leading ribosome. Together, our analyses indicate that in natural mRNA sequences, ribosome collisions arise when proximal ribosomes translate sequences with opposite elongation rates. This combination of sequences sets up a situation whereby a ribosome translating rapidly decoded codons collides with a downstream paused ribosome, thus causing a disome to form and activate quality control pathways (Fig. 5I).

## DISCUSSION

Accurate and efficient mRNA translation is required to generate a healthy and functional proteome. Decades of study have established that the ribosomal decoding of an mRNA proceeds at an uneven speed that is increasingly recognized as playing an instrumental role in determining the subsequent fate of the nascent polypeptide [reviewed in (*76*)]. This mRNA-specific “rhythm of translation” is influenced by multiple factors, including the codon composition, tRNA availability, and the chemical nature of aminoacyl moieties. Here, we determined the relative contribution of these factors to elongation rate in vivo using an unsupervised ML anomaly detection algorithm that circumvents the noisy nature of the data to identify the positions of ribosome pausing. Our analysis across the entire *S. cerevisiae* translatome revealed an interplay between amino acid hydrophobicity and codon optimality regulating elongation rate (Fig. 2I). We further found that these factors exert distinct effects in the different tRNA binding sites of the ribosome and also differs for ribosomes in the pre- or post-accommodation step of the elongation cycle. Our methodology and the insights we uncovered help establish a framework for exploring the impact of ribosome pausing on diverse biological processes and disparate contexts, including in situations where genes of interest are lowly expressed.

In addition to dissecting the relative importance of codon and amino acid features, we also revealed that characteristic nascent chain signatures are associated with pausing at specific tripeptide motifs at the most C-terminal residues of the NC, which remained elusive in previous analyses where no motif clustering was performed (*65*, *66*). These findings suggest that the nascent chain within the tunnel may regulate elongation rate, which resonates with previously reported cases of NC-driven pausing and stalling. For instance, sequences that contain aromatic residues in the exit tunnel caused ribosome stalling in bacteria (*35*, *77*), and polyglutamine-expanded Huntingtin can stall at the boundary with a flanking polyproline tracts (*78*). However, these NC sequences could also play a role in progressing the elongation cycle. Experimental and theoretical studies pose the electrostatic and hydrophobic interactions between the NC and the exit tunnel as modulators of elongation rate (*40*, *66*, *79*). Such interactions might help explain the diversity of charged and hydrophobic residues associated with the different pausing motifs, perhaps by facilitating the progression of the paused ribosome, thus avoiding deleterious ribosome stalling. Future studies should explore the mechanisms by which the NC regulates translation speed, particularly in the tunnel region proximal to the PTC.

Combining our ML pipeline with manipulation of tRNA levels further uncovered the broad and complex interplay between tRNA abundance and wobble pairing on elongation rate. We found that wobble decoding, even when it is mediated by an abundant tRNA, is much slower than direct decoding by a rare cognate tRNA. Thus, the mechanism of tRNA incorporation (i.e., cognate versus wobble pairing) has higher impact on elongation rate than tRNA abundance. Perhaps the most unexpected finding is that even low abundance tRNAs compete for near-cognate codons, resulting in a measurable impact on ribosomal pausing. Thus, altering tRNA levels can have widespread consequences on translation by causing wobble interactions with various noncognate codons. This suggests that the elongation rate of individual codons in an mRNA is determined not only by its individual encoded amino acid and cognate tRNA levels but also by the composition of the tRNA repertoire of the cell that can slow decoding via wobble pairing. These wobble interactions may contribute to the observed low levels of miscoding (*80*) and may also help explain the proteostasis deficits resulting from tRNA manipulation (*81*). Given the nontrivial baseline level of competition between tRNAs, tRNA pools and codon choice must be exquisitely tuned in vivo to achieve the optimal elongation rate at a given position in the mRNA while minimizing deleterious interactions.

Collectively, our findings provide a unified framework of the nuanced mechanisms that dictate how ribosomes ebb and flow along a transcript. Understanding the principles underlying ribosome pausing illuminates key events in cotranslational proteostasis, including NC folding and chaperone and SRP binding, as well as the sequence determinants leading to ribosome collisions in vivo. Our observations raise questions on how the regulation of translation rate constrained the coevolution of proteins as a function of mRNA sequences, the cellular tRNA pool, and the physicochemical and structural features of nascent polypeptides.

## MATERIALS AND METHODS

### Yeast strains

The *S. cerevisiae* BY4741 strain (*MATa his3*Δ*1 leu2*Δ*0 met15*Δ*0 ura3*Δ*0*) was used for obtaining the WT libraries with short and long reads, the high sequencing depth library of long reads (Fig. 1 and fig. S2), and was also the genetic background for the plasmid overexpression of *tRNA^Arg^_CCU_* and *tRNA^Arg^_UCU_*. *S. cerevisiae* Δ*tR(CCU)J::Hyg, MAT*α, *can1*Δ*::MFA1pr-HIS3 mfα1*Δ*::MFα1pr-LEU2 lyp1*Δ *ura3*Δ*0 leu2*Δ*0* was used for obtaining the *∆tRNA^Arg^_CCU_* libraries.

### Data sources and data availability

The list of SRP substrates used in Fig. 1 and fig. S3 was retrieved from the supplementary materials of (*48*) and comes from selective ribosome profiling studies. The TRiC substrates were identified from published selective ribosome profiling data describing TRiC substrates, published in (*49*) and accessed using the Gene Expression Omnibus (GEO) ID GSE114882. The small footprint data used in Fig. 2 corresponds to ribosome profiling data generated using cycloheximide (CHX) and tigecycline, published in (*52*) and retrieved using the GEO ID GSE115162. All other described ribosome profiling experiments were generated as part of this study and have been deposited in the BioProject database under the accession ID PRJNA1039557 and will be made public upon publication. Code for data processing and ribosome pausing identification can be found with the DOI https://doi.org/10.5281/zenodo.12730492.

### Ribosome profiling

To isolate ribosomes, 50 ml of yeast cultures in yeast extract, peptone, and dextrose was grown overnight and used to inoculate 500 ml of cultures at a starting optical density at 600 nm (OD_600_) = 0.05. At an OD_600_ ~ 0.8, cells were harvested by fast filtration and immediately flash-frozen in liquid nitrogen. To lyse the cells, the frozen pellets were combined with 2 ml of lysis buffer [20 mM tris-HCl (pH 7.5), 140 mM KCl, 1.5 mM MgCl_2_, 0.5 mM dithiothreitol, and 1% Triton X-100 (100 mg ml^−1^)] frozen in liquid nitrogen, and grinded in a MM301 mixer mill at 20 Hz for 1 min. Pulverized lysate was thawed in a water bath at room temperature, followed by a centrifugation step at 21,000*g* and 4°C for 20 min. Cleared lysates were digested using ribonuclease I (Ambion) (RNA concentration was measured for the cleared lysates and used to determine the amount of needed enzyme) at room temperature for 25 min, and the reaction was stopped by adding SUPERase^.^In (Ambion). Ribosomal pellets were recovered from the digested lysates using a 25% (w/v) sucrose cushion prepared in lysis buffer, without Triton X-100 and CHX, and centrifuged at 72,000*g* and 4°C for 25 min. Total RNA was extracted from the pellets using the hot SDS-phenol-chloroform method. For libraries containing short and long reads, RNA footprints on the 17- to 34-nt range were isolated, whereas for long-reads only libraries, the 24- to 34-nt range was used. Ribosome profiling libraries were prepared as previously described (*82*). After quantitative polymerase chain reaction quantification (Kapa Biosystems), libraries were sequenced using HiSeq 4000 (Illumina).

### Data processing

Sequencing data were demultiplexed using the CASAVA suite (Illumina), followed by the removal of the added Universal miRNA cloning linker using Cutadapt (*83*). To avoid low-quality bases, the first nucleotide on the 5′ end was removed using Cutadapt. Trimmed libraries were mapped against an *S. cerevisiae* ribosomal RNA (rRNA) index using bowtie1 (*84*). Reads that did not align to rRNA were mapped against the *S. cerevisiae* transcriptome using TopHat (*85*) with bowtie1 as aligner option. Both rRNA and transcriptome indices were built on the basis of the R64-1-1 Ensembl genome assembly for *S. cerevisiae* (*86*). Only reads that mapped unambiguously to the transcriptome, with zero mismatches, were used for further analysis. Aligned libraries were annotated at the codon level using the RiboProfiling Bioconductor package (*87*) under the following criteria: Only read lengths displaying a clear 3-nt periodicity were kept, and an individual P-site offset was estimated on the basis of the initiation codon.

### Ribosome pausing identification

A normalized translation profile for each transcript in an annotated library was obtained by normalizing the raw number of read counts per codon by the mean read counts per transcript. High values in such a normalized profile (i.e., outliers) represent codons at which translation takes longer to occur and will be referred to from here on as pausing peaks. However, outlier identification is relative to the expression level of each transcript: Outliers in highly expressed genes will exhibit higher read values than outliers in poorly expressed genes. To account for that, transcripts per kilobase million (TPM) were calculated for each annotated library (dataset) and used as a proxy to classify the ORFs in each sample according to their expression level. The distribution of TPM values was binned in five intervals: (0 to 20%), [20 to 40%), [40 to 60%), [60 to 80%), and [80 to 100%]. This classification was used for the identification of “pausing peaks” within similarly expressed genes, thus avoiding considering the entire dataset at once and therefore annotating only pausing peaks of highly expressed genes. Another consideration for accurate outlier identification in ribosome profiling data has to do with the inherent higher coverage at the beginning and end of the sequenced ORFs, due to higher ribosome densities coming from translation initiation and termination. To mitigate this, both the normalized read counts and the normalized codon position associated with each read count number have to be accounted for. Pausing peaks were then identified in two-dimensional data (normalized counts versus normalized codon position) using EIF models (*42*). EIF is a powerful unsupervised learning tool for anomaly detection in multidimensional data, working on the principle of anomaly isolation rather than the characterization of the normal instances of a dataset (*43*). An EIF model based on the two abovementioned features, normalized counts and position, was generated for each of the defined TPM intervals using the R package “eif” (*42*). Each forest was composed of 200 trees, and the sample size to build each tree was set to be the mean of the length of all ORFs in each TPM interval. After the EIF was generated, the dataset was run through the model to obtain the corresponding anomaly scores for each codon. Codons whose score fell in the 0.95 quantile were labeled as pausing peaks. Further relaxation of this threshold, or increased stringency, does not seem to affect the distribution of enrichments in the identified pausing peaks (fig. S1C).

The EIF hyperparameters were set on the basis of the original isolation forest work (*88*). Maximum depth is defined by the algorithm itself, it being ceiling[log_2_(subsampling size)] (*88*). The idea behind this definition is that the isolation of anomalies using this branching procedure focuses on nodes with less-than-average path lengths (i.e., anomalies are isolated faster than normal points). In our case, we set the subsampling size to be the mean ORF length in each TPM, which allows for this to vary according to the number of training points. The original Isolation Forest study characterized an optimal of 256 subsampling size for a variety of applications, showing negligible AUC gains beyond that point (*88*). Our subsampling size strategy results in values >350, reasonably well above the suggested default. The number of trees was set to a high number (200) based on the original Isolation Forest publication, which shows how the ensemble stabilizes well before 100 trees (*88*). To evaluate this in the context of ribosome profiling signals with biologically relevant read distributions, we decided to place known pausing points, i.e., outliers, in actual RiboSeq data. For this, we used previously published data (*47*) to avoid reanalyzing the one from this study. First, we identified all positions whose normalized read count was greater than 2 SDs, as estimated for their corresponding ORFs. We then converted all those positions to the mean value of the corresponding ORF. This procedure harshly eliminates all extreme values from the read distribution. Then, we added a random value between 2.5 and 3 SDs to the normalized read counts of random positions in each ORF, thus creating a series of pausing points (outliers) we can use as ground truth. Last, we applied our pipeline to these data and monitored performance with the AUC. The classification task performs very well with the parameters used in this study, trees = 200, subsample size = mean (ORFs length) (fig. S1). This is not unexpected as those values are well above the suggested defaults for the isolated forest search (*88*). Furthermore, by varying either the number of trees (fig. S1A) or the subsample size (fig. S1B) and keeping fixed the other hyperparameter at the proposed value, we can see that the task that appears is very robust across a wide range of values. This implies that those numbers can be safely increased, but the performance gain will most likely be marginal compared to the computing time.

The conventional ribosome pausing estimates displayed in Fig. 1B were estimated as position specific *z* scores. Thus, the mean and SD used in the *z* score estimation of position *i* of a given ORF belonging to a specific TPM interval originate from the distribution of normalized reads at all *i*th positions of all ORFs in such TPM interval, as also defined for the EIF models. The last 10 codons of each ORF were excluded as not all ORFs have the same length, thus avoiding including stop codons in the read distributions.

### Codon optimality scale

The codon optimality definition used in this study is the classical translation efficiency scale for *S. cerevisiae* as published in (*6*) and was retrieved from the supplement of the study. The tAI was used as originally defined by dos Reis *et al.* (*89*), implemented in the tai R package.

### Logo plot analysis and tripeptide clustering

The information content values, *H*, for the logo plots were calculated using an ungapped multiple sequence alignment of the residues in the PTC (Figs. 3, D, E, and G, 4A, and 5G) or the 28 residues upstream of the PTC (Fig. 4A) when a pausing peak is in the P site of the PTC. *H* at position *l* of the alignment was computed as described in (*90*) using the equation

$$H_{l}=\sum_{i\in A}H_{\left(l,i\right)}=\sum_{i\in A}\left(q_{\left(l,i\right)}{\text{log}}_{2}\frac{q_{\left(l,i\right)}}{p_{i}}\right)$$

where *A* is the set of the 20 common amino acids; *q*_(*i*,*l*)_ is the fraction of amino acid *i* at position *l*; *p_i_* is the probability of observing amino acid *i* in the proteome of interest. Negative *H*_(*l*,*i*)_ values indicate that the frequency of amino acid *i* at position *l* is less than expected, while positive values indicate amino acid enrichment. The estimated *H*_(*l*,*i*)_ values were plotted using ggseqlogo (*91*). Pausing motifs depicted in Fig. 4 were created by identifying the significantly enriched tripeptide motifs, where the central residue is at the P site and is a pausing peak, and grouping them using hierarchical clustering based on restricted Damerau-Levenshtein distance.

### Secondary structure annotation

Secondary structures for the proteins of the *S. cerevisiae* proteome were predicted using s2D software (*92*).

### Enrichment analysis

Enrichment values in this study are odds ratios estimated using a Fisher’s exact test, and their statistical significance is given by the associated *P* value from the test. Benjamini and Hochberg *P* value adjustment was used for multiple hypothesis correction. All statistical testing was performed in the R environment (http://r-project.org).
