# Supplementary material for: A machine learning approach uncovers principles and determinants of eukaryotic ribosome pausing
Source: Sci Adv. 2024 Oct 18;10(42):eado0738. doi: 10.1126/sciadv.ado0738 (PMC11488575; doi:10.1126/sciadv.ado0738)
Supplement: Supplementary file 1 — Figs. S1 to S5 [file sciadv.ado0738_sm.pdf]

Science Advances
AAAS

Supplementary Materials for

A machine learning approach uncovers principles and determinants of eukaryotic ribosome pausing

Mauricio Aguilar Rangel *et al.*

Corresponding author: Judith Frydman, jfrydman@stanford.edu

Sci. Adv. **10**, eado0738 (2024)
DOI: 10.1126/sciadv.ado0738

This PDF file includes:

Figs. S1 to S5

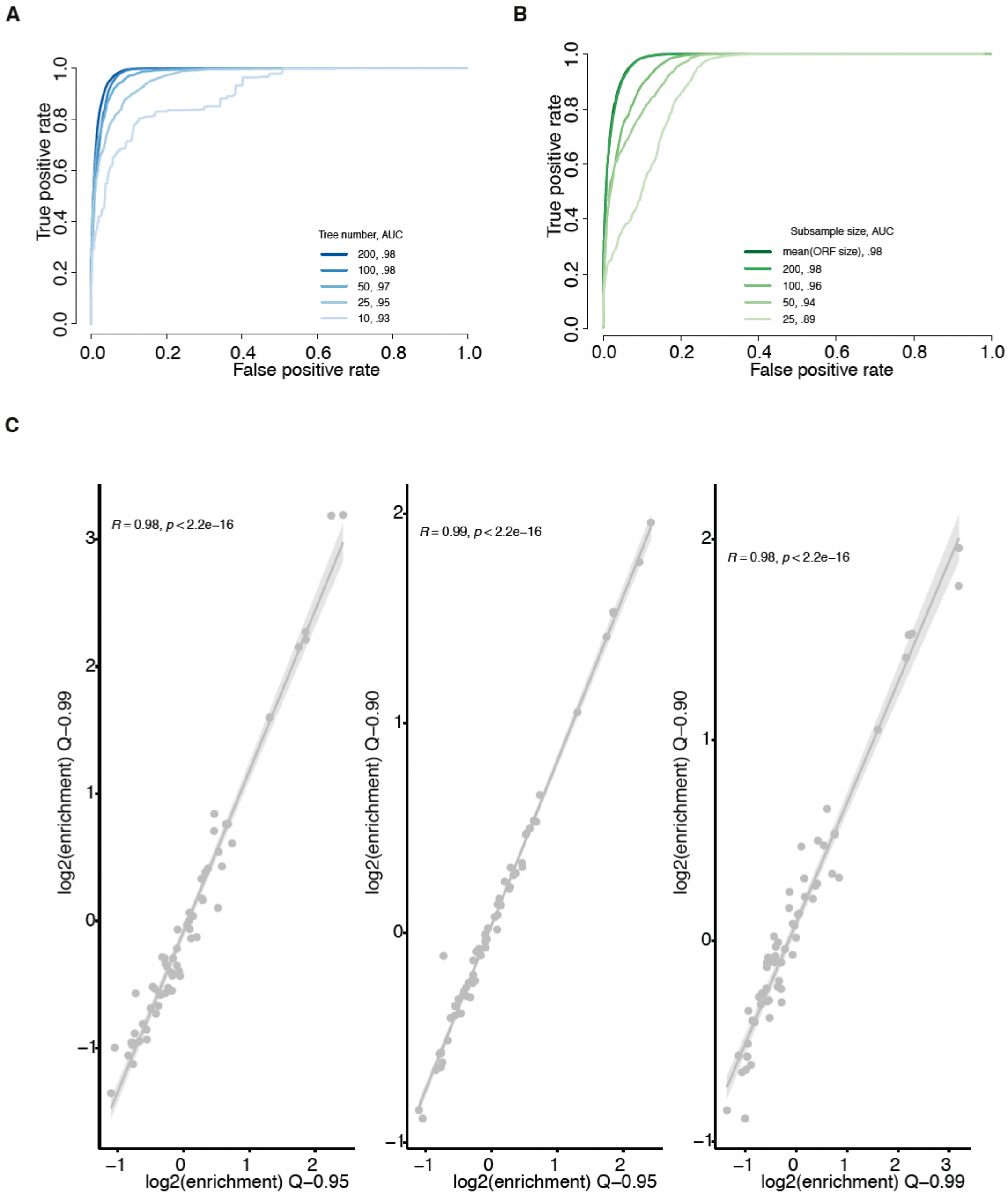

Fig. S1. Effects of hyperparameter variation in model performance. (A) ROC plots corresponding to different tree size numbers used in simulated data. (B) ROC plots corresponding to different sub-sample size numbers used in simulated data. (C) Cross-comparison of codon enrichments at identified pausing positions using three different quantile thresholds. Pearson correlation coefficient in inset.

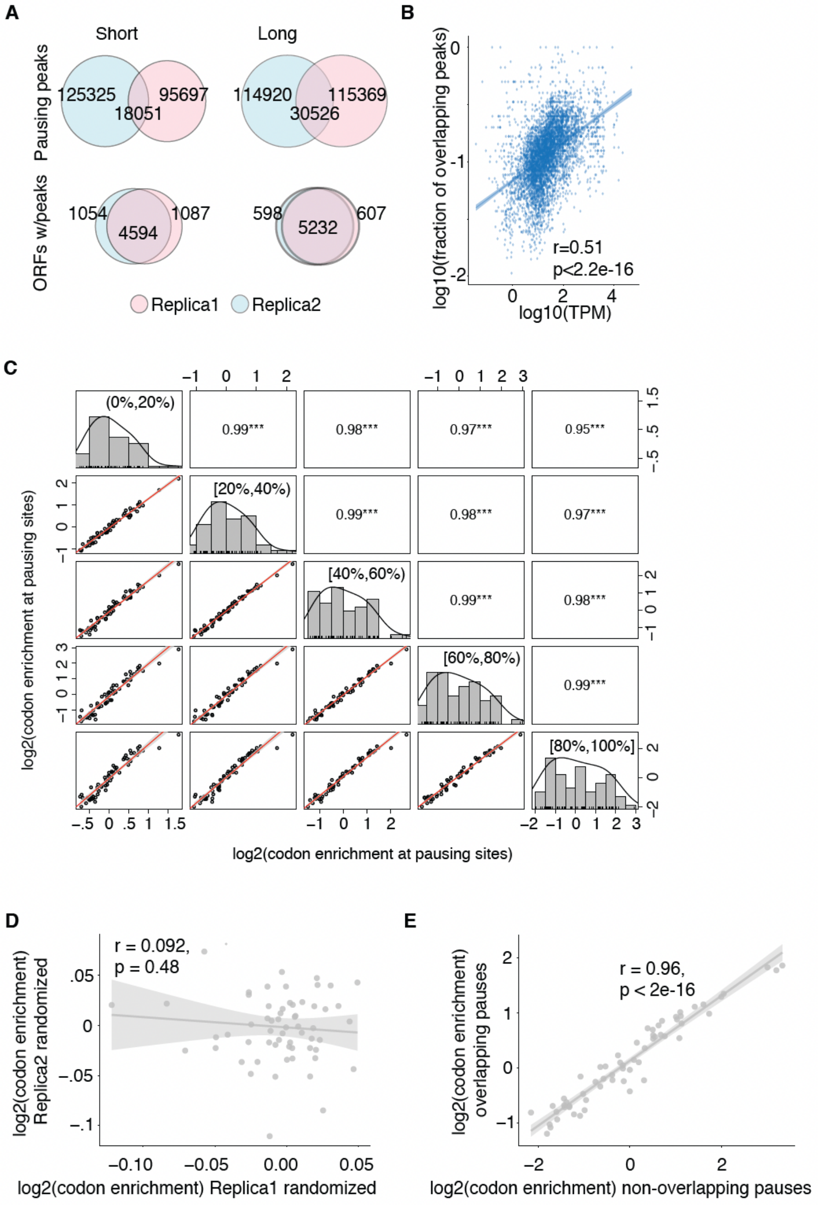

Fig. S2. Statistics on identified pausing sites. **(A)** Venn diagrams illustrating the overlap of identified pausing peaks (top) and open reading frames containing pausing sites (bottom) between two biological replicates. **(B)** Correlation plot between gene translation level measured in transcripts per million (TPM) and the fraction of overlapping pausing sites between replicates (each point represents an ORF). **(C)** Cross-correlation of the observed codon enrichments at pausing sites at every TPM quantile used during pausing identification (all vs all comparison; TPM quantile noted atop each histogram). **(D)** Correlation plot of the estimated enrichment of individual codons (log2) in a random set of codon positions in two separate replicates (same number of positions as the number of identified pausing peaks in those sequencing libraries). **(E)** Correlation plot between the enrichment of individual codons in the overlapping and non-overlapping pausing peaks of two biological replicates.

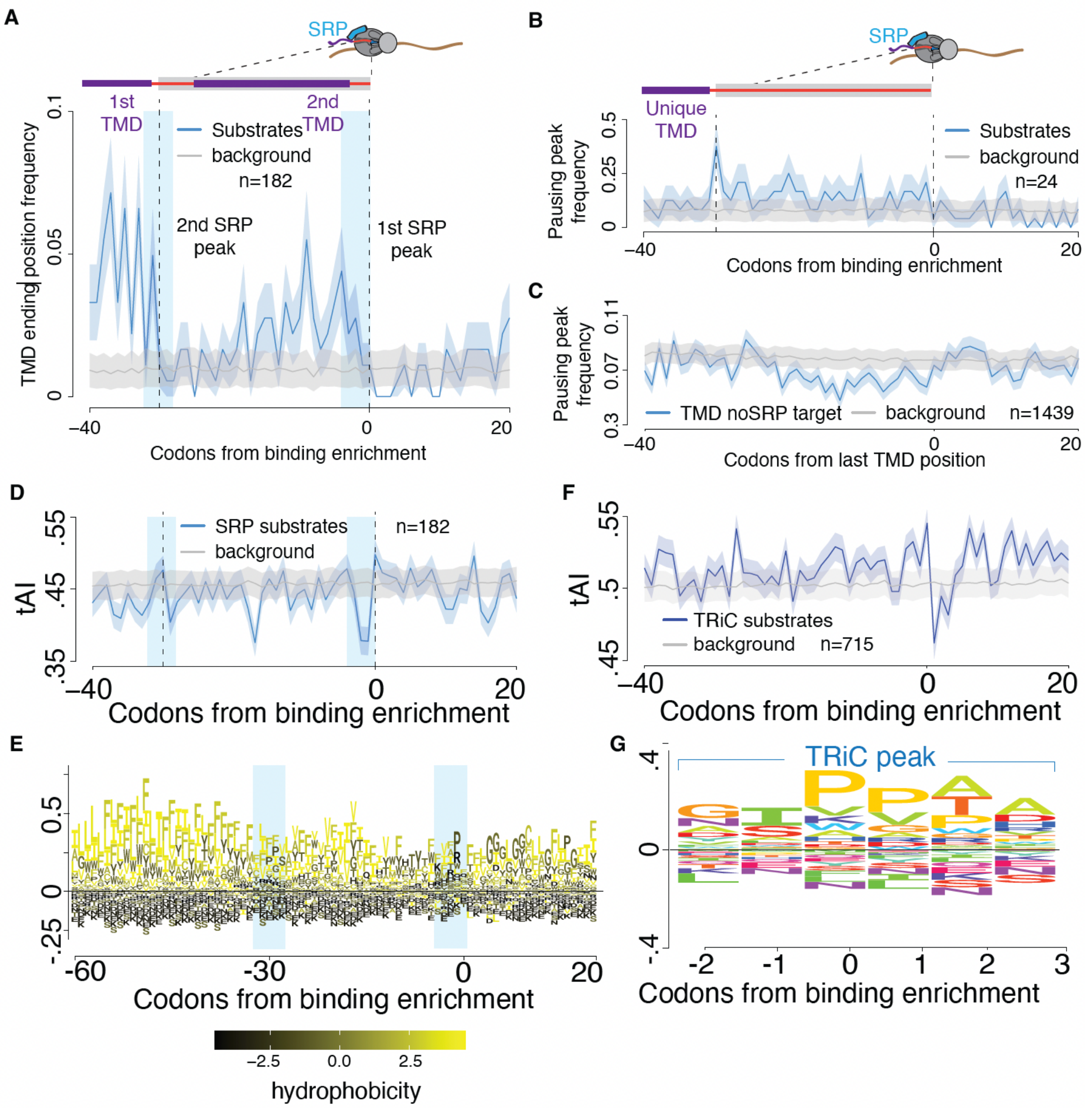

Fig. S3. Correlation of cotranslational chaperone binding events and ribosome pausing. (A) Metagene plot showing the frequency of C-terminus ending positions of transmembrane domains (TMD) for the signal recognition particle (SRP) chaperone substrates, at the moment of binding between the SRP and the nascent chain. Light blue shaded areas correspond to the binding events of SRP (Fig. 1G). **(B-C)** Metagene analysis of pausing peak frequency at the moment of SRP binding to the nascent polypeptide chain for (B) substrates containing a single TMD, or (C) proteins containing TMD that are not targets of SRP. Background is estimated from random positions in the yeast proteome. **(D,F)** Metagene analysis of the translation adaptation index (tAI) values for the SRP (D, two binding events shaded blue) and TRiC (F) substrates, centered at the peak of chaperone-nascent chain recognition. **(E,G)** Logo plots highlighting the amino acid enrichment for the SRP (E, two binding events shaded blue) and TRiC (G) substrates at the moment of chaperone binding. In all panels negative numbers indicate translated codons, and positive ones the yet to be translated ones.

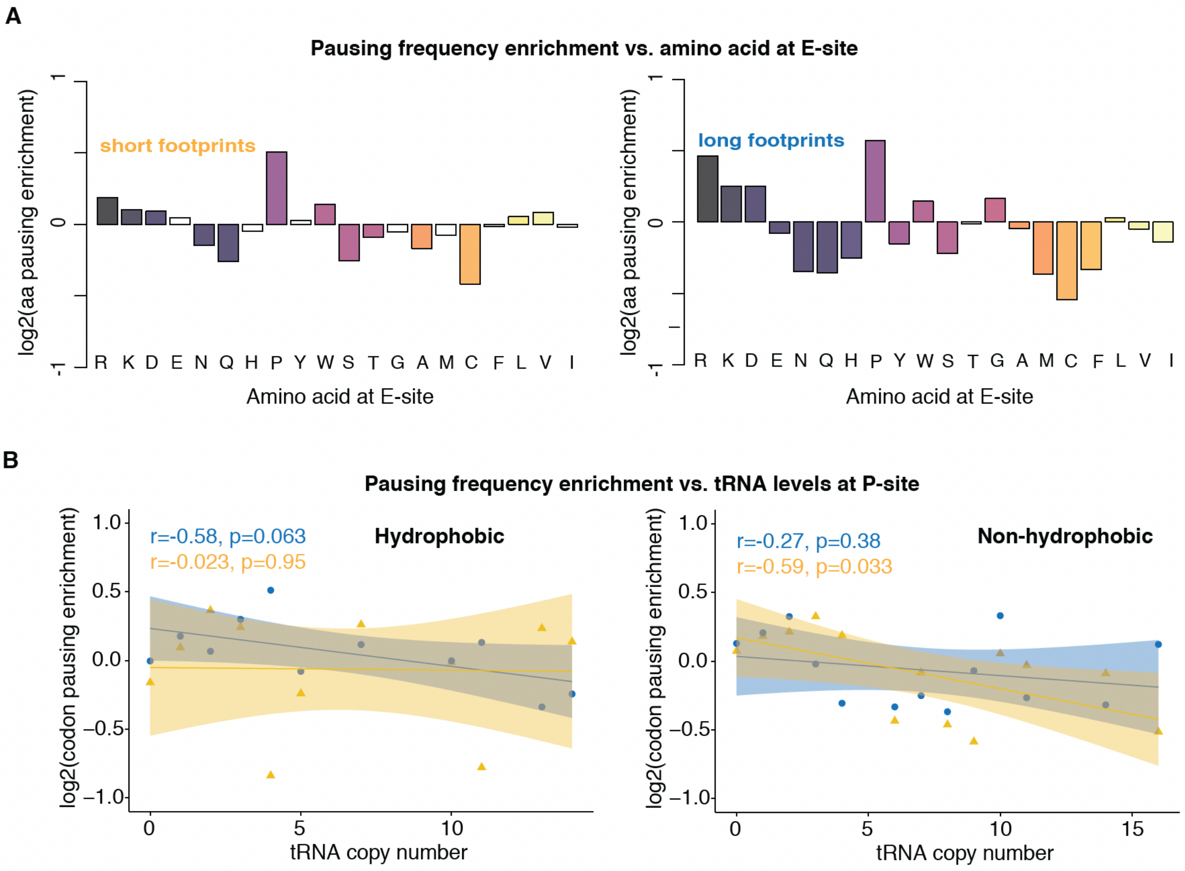

Fig. S4. Amino acid and codon optimality interplay during ribosome pausing. (A) Amino acid enrichment analysis in the identified pausing peaks, E-site centered, separated by replicate of short (left) and long (right) reads. **(B)** The correlation between the enrichment of the codons in the P-site when a ribosome pausing event is mapped to the A-site, and their corresponding number of decoding tRNA gene copies, separated according to amino acid hydrophobicity.

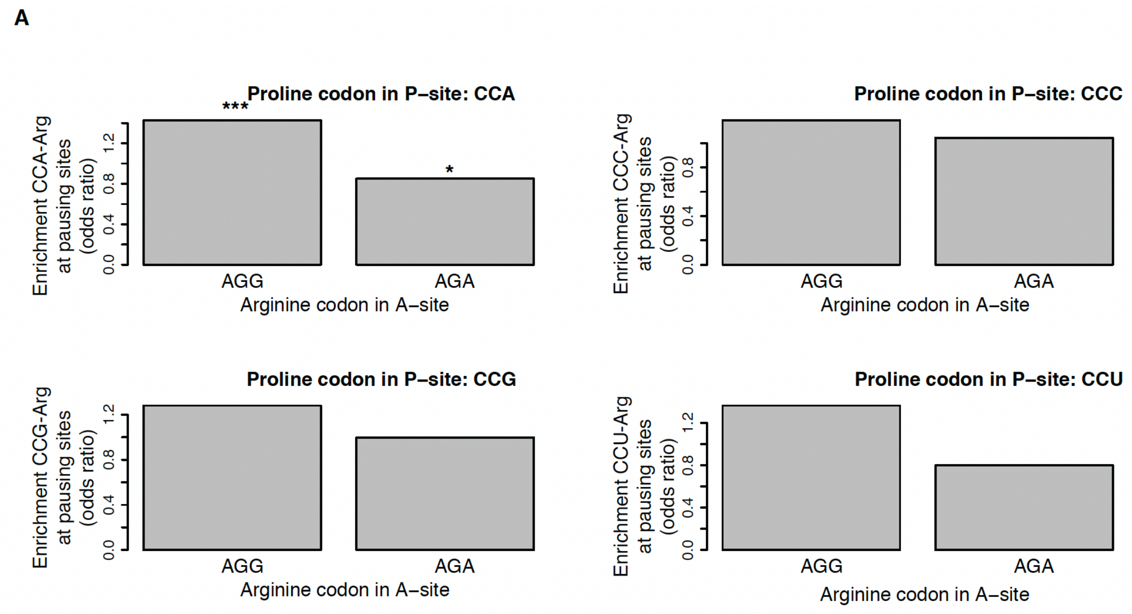

Fig. S5. Enrichment analysis of CC(A/C/G/U)-AG(A/G) codon pairs at the respective CC(A/C/G/U) pausing sites. Pausing sites correspond to those identified in the S. cerevisiae empty background described in Fig. 2F and H. Asterisks indicate statistical significance of the odds ratio (Fisher's exact test).
